# A pre-illness reference reminder alters cross-sectional patient-reported outcome responses: evidence from EQ-5D-5L and EQ-HWB-9

**DOI:** 10.1186/s12955-026-02580-2

**Published:** 2026-07-03

**Authors:** Xun Ran, Qimeng Mu, Qiaomei Wang, Zhihao Yang

**Affiliations:** 1 Department of Clinical Medicine, Guizhou Medical University, Guiyang, China; 2 Department of Health Services Management, Guizhou Medical University, Guiyang, China

**Keywords:** Response shift, Recalibration, Patient-reported outcome measures, Cross-sectional study

## Abstract

**Objectives:**

This study aimed to evaluate whether a brief pre-survey reminder prompting patients to reflect on their pre-illness health was associated with altered response patterns in cross-sectional patient-reported outcome measures (PROMs).

**Methods:**

A cross-sectional study was conducted among 244 inpatients in a hepatobiliary surgery department. Participants were assigned to a control group (standard recall instructions for the EQ-5D-5L and EQ-HWB-9) or a reminder group (identical instructions, preceded by the reminder). Response distributions were compared descriptively, and adjusted ordered logistic regression models were used to assess the association between the reminder and reported problem severity.

**Results:**

A total of 244 inpatients participated, with 123 in the control group and 121 receiving the pre-illness reminder. There were no missing data for the EQ-5D-5L or EQ-HWB-9 items, and EQ VAS data were missing for three participants only. The reminder group reported lower ceiling effects and higher problem severity across both instruments. In adjusted analyses, the reminder was associated with higher odds of reporting more severe problems across all EQ-5D-5L dimensions, with odds ratios ranging from 1.90 to 3.13, and across seven of the nine EQ-HWB-9 items, with odds ratios ranging from 1.94 to 5.43. The reminder group also reported a lower mean EQ VAS score than the control group: 78.23 versus 82.03, *p* = 0.002.

**Conclusions:**

The brief reminder was associated with more severe and differentiated PROM responses. These findings suggest that a pre-illness reference reminder may influence respondents’ appraisal of current health in cross-sectional PROM assessment. However, without an objective benchmark for true health status, the findings should be interpreted as consistent with, but not definitive evidence of, a recalibration-related response process.

## Highlights

This study tested a brief interviewer-delivered pre-survey reminder prompting patients to reflect on their pre-illness health before completing standard PROMs.

The reminder was associated with lower ceiling effects and greater reporting of problem severity across the EQ-5D-5L and EQ-HWB-9.

The findings suggest that pre-illness reference reminders may influence PROM response patterns, although reduced recalibration cannot be distinguished from increased symptom salience, framing effects, or other response processes.

## Introduction

Patient-reported outcome measures (PROMs) are widely used to assess health status and health-related quality of life from the patient’s perspective [1, 2]. Instruments such as the EQ-5D are routinely applied in clinical studies, health services research, and economic evaluations, and are often treated as straightforward reflections of an individual’s current health [3, 4]. However, accumulating evidence suggests that PROM responses are not only determined by underlying health status, but also by how individuals interpret, evaluate, and contextualize their health when answering questionnaire items [5, 6].

A key challenge in interpreting PROM data is the phenomenon known as response shift [7]. Response shift refers to a change in the meaning of an individual’s self-evaluation of a target construct, such as health or quality of life [8]. This can occur through three main mechanisms: recalibration, where internal standards of measurement change; reprioritization, where the relative importance of different health domains shifts; and reconceptualization, where the underlying concept of health itself is redefined [7, 9]. As a result, two individuals with similar health states—or the same individual at different points in time—may report different outcomes not because their health differs, but because their frames of reference differ.

Although response shift is often discussed in longitudinal studies [10, 11], it is increasingly recognized that it also poses a substantial problem in cross-sectional research. In cross-sectional settings, different respondents may use fundamentally different internal standards when rating their health, particularly when comparing people with and without illness, or people at different stages of disease adaptation [12]. For example, individuals living with chronic conditions may adapt to their symptoms and adjust their expectations of what constitutes “good” or “poor” health [13]. As a consequence, cross-sectional comparisons may underestimate the burden of illness or obscure true differences between patient groups and the general population.

Generic PROMs such as the EQ-5D may be particularly susceptible to recalibration effects in cross-sectional analyses. The EQ-5D explicitly uses a “today” recall period [14, 15], which encourages respondents to focus on their current functioning and symptoms. While this design minimizes memory bias, it does not prevent respondents from implicitly adjusting their internal standards based on past experiences, adaptation to illness, or changes in expectations. Two respondents answering “today” may therefore be using very different benchmarks when deciding what constitutes “no problems,” “some problems,” or “extreme problems.”

Existing methodological approaches to response shift have primarily focused on detection rather than prevention [16–18]. Many methods rely on longitudinal data, retrospective assessments, or complex statistical modeling to identify response shift after it has occurred [11, 19]. These approaches are often infeasible in routine data collection, add analytical complexity, or are unsuitable for cross-sectional studies [20]. Importantly, relatively little attention has been paid to simple, low-burden design strategies that might influence respondents’ frames of reference at the point of data collection.

In this study, we propose a novel and pragmatic approach aimed at examining whether a pre-illness reference reminder influences PROM response patterns. Rather than altering the official recall period of the instrument, we introduce a brief instructional reminder prior to questionnaire completion. Respondents are asked to think about their symptoms and reflect on how their health was when they were not ill, before completing the PROM using the standard “today” recall period. This approach preserves the original instrument structure and recall period, while explicitly making a pre-illness reference point salient during questionnaire completion.

Conceptually, this intervention seeks to influence the appraisal process underlying PROM responses by standardizing the frame of reference across respondents. By making the pre-illness state salient, the reminder is intended to examine whether making pre-illness health salient is associated with different reporting patterns, particularly among patients who have adapted to chronic symptoms or functional limitations. Unlike retrospective pretests or alternative recall periods, this approach does not ask respondents to re-rate past health, nor does it replace contemporaneous self-assessment. Instead, it subtly guides respondents toward a more consistent comparison standard while maintaining focus on current health.

The present study evaluates the impact of this pre-survey reminder in a cross-sectional setting, with particular attention to whether it alters response distributions and improves the interpretability of PROM data. By examining a simple design feature that may influence response appraisal, this study contributes a novel perspective to the response shift literature and offers a potentially scalable strategy for improving the validity of cross-sectional patient-reported outcome measurement.

## Methods

### Study design and setting

Data were collected between July and August 2025 in the Hepatobiliary Surgery Department of the Affiliated Hospital of Guizhou Medical University, China. Two independent samples of inpatients were recruited during routine hospitalisation. Patients in the control group completed the questionnaire using the standard recall instruction (“today”), while patients in the intervention group received an additional instruction prior to questionnaire completion, asking them to think about their symptoms and how their health was when they were not ill, before responding using the same “today” recall period. Apart from this instructional difference, questionnaire content and administration procedures were identical across groups.

The study was approved by the institutional ethics committee of Guizhou Medical University. All participants provided informed consent prior to participation, and all data were anonymised before analysis.

### Participants and data collection

Eligible participants were adult inpatients admitted to the Hepatobiliary Surgery Department of the Affiliated Hospital of Guizhou Medical University. The two study arms were recruited sequentially: the control arm was collected in July 2025, and the reminder arm was collected in August 2025. In both arms, eligible patients were first identified and recruited by a clinician. A trained interviewer then approached the patient, explained the study, obtained informed consent, and invited the patient to complete the questionnaire by self-report on a tablet. The questionnaire was programmed and administered using Wenjuanxing (www.wjx.com), a widely used online survey platform in China. No financial or other incentives were provided. The same department, eligibility criteria, and overall data collection workflow were used for both arms. The control arm did not receive any further instructions beyond the standard questionnaire instructions. In the reminder arm, before participants completed the questionnaire, the interviewer orally instructed them: “Please first tell me about your current symptoms due to the health condition. When answering these questions, please think about your health when you did not have this health condition.” Clinical information, including primary diagnosis, main complaints at admission, and history of chronic conditions, was retrieved from the hospital information system by a trained research assistant. These variables were extracted independently of the questionnaire responses to minimise respondent burden and reporting bias. Diagnoses, main complaints, and chronic conditions were subsequently categorised for analysis using a predefined, rule-based approach, as described below.

The survey collected patient-reported outcome measures and background sociodemographic information, including age, sex, education level, household income, and place of residence.

### Measures

Health-related quality of life was assessed using the EQ-5D-5L descriptive system and the EQ visual analogue scale (EQ-VAS) [21]. The EQ-5D-5L comprises five dimensions—mobility, self-care, usual activities, pain/discomfort, and anxiety/depression—each with five ordered response levels ranging from no problems to extreme problems. The EQ VAS records self-rated health on a scale from 0 to 100, with higher scores indicating better perceived health.

In addition, wellbeing was assessed using the EQ-HWB-9 [22, 23], a set of nine items designed to capture a broader range of physical, cognitive, and psychosocial aspects of health and wellbeing. The EQ-HWB-9 items assess usual activities, mobility, exhaustion, loneliness, difficulty thinking clearly, anxiety, sadness or depressed mood, perceived lack of control over daily life, and physical pain. Each item uses a five-level ordered response scale referring to the previous seven days, with higher levels indicating greater severity or frequency of problems. Both the EQ-5D-5L and EQ-HWB-9 were included to examine whether the reminder-related response pattern was consistent across PROMs with different descriptive scope, item content, and recall periods. This allowed us to assess whether the observed pattern was instrument-specific or more broadly applicable across measures. Note that the intervention did not alter the official recall period or item wording of either instrument. Participants completed the EQ-5D-5 L using its standard “today” recall period and the EQ-HWB-9 using its standard “previous seven days” recall period. The reminder was communicated orally only as a brief pre-survey instruction before questionnaire completion.

No formal effect-size-based sample size calculation was conducted because no prior estimate was available for the effect of a pre-illness reference reminder on EQ-5D-5L or EQ-HWB-9 responses. The study was therefore designed as an exploratory PROM methodology study. The target sample size was determined pragmatically with reference to common PROM measurement-property studies, with COSMIN guidance used as a benchmark for subgroup comparisons.

### Data organization

To support consistent coding and adjusted comparisons between the two study samples, clinical information on diagnoses, main complaints, and history of chronic conditions was standardised prior to analysis. These variables were originally recorded as free-text entries in the hospital information system and were therefore heterogeneous in wording and level of detail. A predefined, rule-based keyword approach was used to recode these data into a small number of clinically meaningful categories with sufficient sample sizes in each group.

Primary diagnoses were grouped into three mutually exclusive categories reflecting major clinical pathways in hepatobiliary surgery: biliary stone or inflammatory disease; hepatobiliary–pancreatic structural disease; and other or non-hepatobiliary conditions. The structural disease category included hepatobiliary malignancies or mass lesions, pancreatic diseases, and chronic liver diseases, which were combined due to their relatively low frequency and comparable clinical severity. A hierarchical priority rule was applied to ensure that each participant was assigned to a single primary diagnosis category, thereby avoiding overlap across groups.

Main complaints at admission were categorised based on the dominant presenting symptom. Given the high prevalence of pain-related presentations, complaints were dichotomised into abdominal pain–dominant versus non–abdominal pain–dominant symptoms. Abdominal pain–dominant complaints included abdominal, upper abdominal, or right upper quadrant pain, while non–abdominal pain–dominant complaints included jaundice-related symptoms, infection-related systemic symptoms, post-procedural complaints, and other non-specific presentations. This dichotomisation was adopted to balance group sizes across study arms and to minimise sparse categories.

History of chronic conditions was dichotomised into presence versus absence of any chronic condition. Chronic conditions included, but were not limited to, hypertension, diabetes, cardiovascular disease, chronic liver disease or viral hepatitis, tuberculosis, cancer history, and chronic mental health conditions. This binary classification was used to improve statistical stability and to facilitate straightforward adjustment for baseline health status when comparing outcomes between the two samples.

### Data analysis

Baseline characteristics of the control and intervention groups were summarised descriptively and compared using formal statistical tests. Categorical variables were summarised as frequencies and percentages and compared using chi-square tests.

Response distributions for each EQ-5D-5L dimension and each wellbeing item were examined descriptively using stacked bar charts, stratified by study arm. Differences in response distributions between the two groups were formally assessed using Fisher’s exact tests, given sparse cell counts are expected at higher severity levels.

To assess whether the pre-survey reminder was associated with differences in reported problem severity after adjustment for covariates, ordered logistic regression models were fitted separately for each EQ-5D-5L dimension and each wellbeing item. Due to sparse responses in the highest severity categories, response levels 3, 4, and 5 were collapsed into a single category prior to modelling. Outcomes were therefore specified as three-level ordinal variables: no problems, slight problems, and moderate to extreme problems.

All models included study arm as the primary independent variable and were adjusted for age, sex, education, diagnosis category, main complaint category, and chronic condition status. Odds ratios (ORs) and standard errors (SEs) are reported, with an OR greater than 1 indicating higher odds of reporting more severe problems. Model fit was assessed using the Nagelkerke pseudo-R². All analyses were conducted using Stata 18.

## Results

A total of 244 respondents participated in the survey, with 123 assigned to the control group and 121 to the intervention group. For the demographic and clinical characteristics, there were four missing values for surgery status in each group, and three and one for chronic conditions in the control and intervention groups, respectively. Therefore, percentages for these variables were calculated using non-missing observations. There were no missing data for the EQ-5D-5L or EQ-HWB-9 items in either study arm. EQ VAS data had three missing values in total, including one in the control group and two in the intervention group. All available data were used in the corresponding analyses.

Table 1 summarises the demographic and clinical characteristics of participants by study arm. The control (*n* = 123) and intervention (*n* = 121) groups were broadly comparable across all measured variables. There were no statistically significant differences between the two arms in sex, age group, educational attainment, diagnosis category, chief complaint, surgery status, or history of chronic conditions (all *p* > 0.05). In both groups, biliary stone or inflammatory disease was the most common diagnosis, and abdominal pain–dominant symptoms accounted for the majority of presenting complaints. These findings suggest that the two groups were similar with respect to the observed baseline characteristics included in the analysis.

**Table 1:** Characteristics of the sample, by arm

		Arm 1: Control, *n* = 123		Arm 2: Reminder, *n* = 121		
		n	%	n	%	
Sex	Male	56	45.53	59	48.76	*P* = 0.613
	Female	67	54.47	62	51.24	
Age group	<=40	22	17.89	25	20.66	*P* = 0.632
	41–60	48	39.02	51	42.15	
	> 60	53	43.09	45	37.19	
Education	Middle school or below	80	65.04	83	68.60	*P* = 0.672
	High school / vocational school	19	15.45	14	11.57	
	College / university (associate’s or bachelor’s degree)	24	19.51	24	19.83	
Diagnosis	Biliary stone / inflammation	74	60.16	78	64.46	*P* = 0.237
	Structural disease (malignancy/mass OR pancreas OR chronic liver)	36	29.27	25	20.66	
	Other / non-hepatobiliary	13	10.57	18	14.88	
Chief complaint	Abdominal pain–dominant	79	64.23	69	57.02	*P* = 0.250
	Non–abdominal pain dominant	44	35.77	52	42.98	
Surgery status	No	69	57.98	69	58.97	*P* = 0.877
	Yes	50	42.02	48	41.03	
Chronic conditions	No	66	55.00	76	63.33	*P* = 0.189
	Yes	54	45.00	44	36.67	

Note. Percentages were calculated using non-missing observations for each variable. Missing values were present for surgery status (Arm 1: *n* = 4; Arm 2: *n* = 4) and chronic conditions (Arm 1: *n* = 3; Arm 2: *n* = 1) and are reported in the table where applicable

**Fig. 1:**
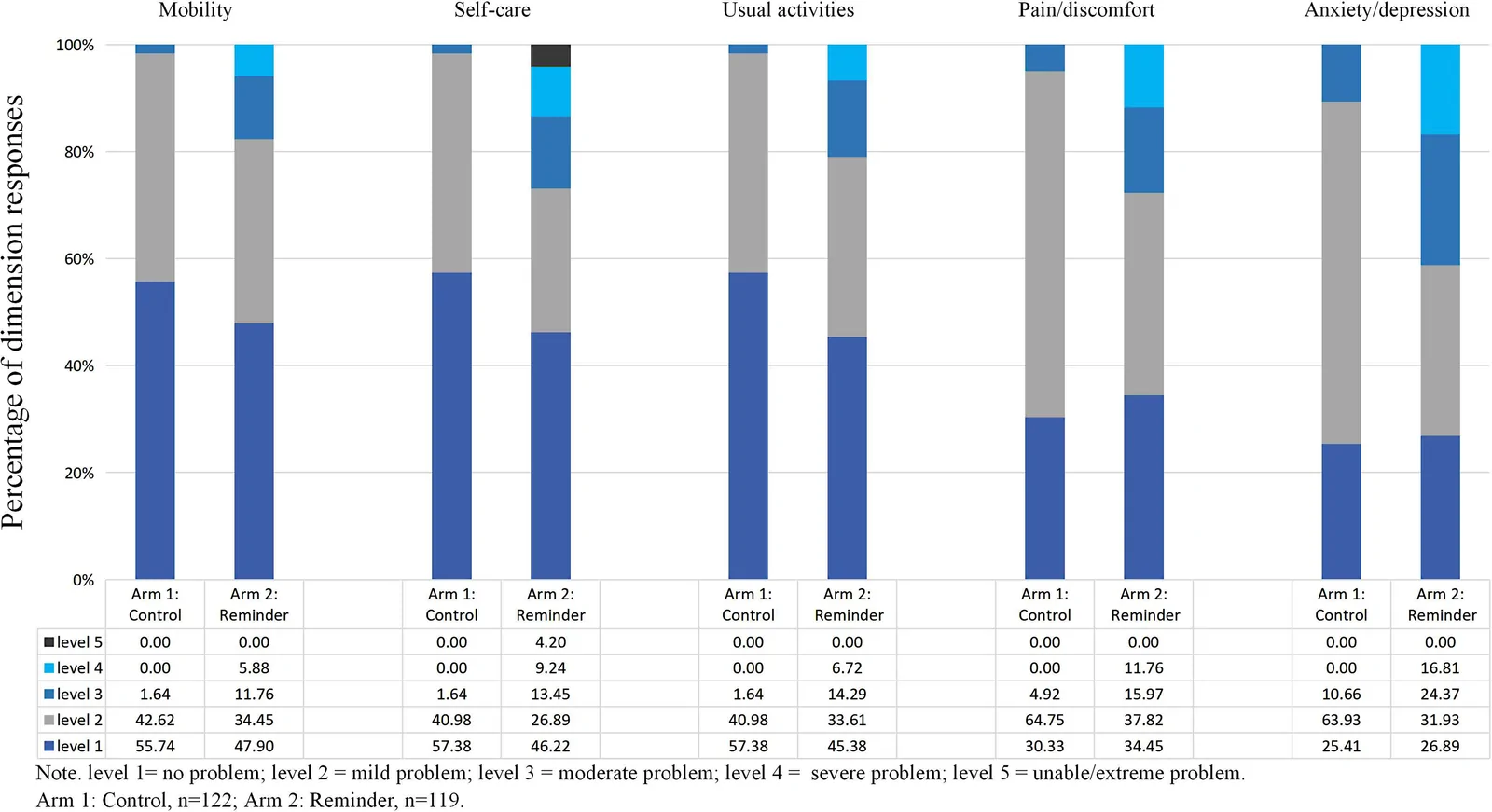
EQ-5D-5L responses between two arms

Figure 1 compares the response distributions of the EQ-5D-5L dimensions between the control group (Arm 1, standard recall period of “today”) and the intervention group (Arm 2, symptom recall with reference to a healthy time).

Across mobility (MO), self-care (SC), usual activities (UA), more than half of responses in the control group are at level 1, indicating ceiling effects under the standard “today” recall instruction. Notably, no responses are observed at levels 4 or 5 in any dimension in Arm 1, suggesting limited use of the upper severity levels.

In contrast, the intervention group shows a clear redistribution of responses away from level 1 toward higher severity levels. The proportion of level 1 responses decreases across the dimensions of mobility, self-care, and usual activities, accompanied by increased reporting at levels 3, 4, and 5 across all five dimensions. Responses at levels 4 and 5—absent in the control group—are consistently observed in the intervention group, with the largest shifts seen in pain/discomfort and anxiety/depression. Fisher’s exact tests showed that response distributions differed significantly between the control and reminder arms for all EQ-5D-5L dimensions (all *p* < 0.01).

In addition to the dimension-level response distributions, a similar pattern was observed for the EQ VAS. The mean EQ VAS score in the control group (Arm 1, standard “today” recall) was 82.03 (SD 6.03), whereas the intervention group (Arm 2, symptom recall with reference to a healthy time) reported a lower mean score of 78.23(SD 12.08). The difference between groups was statistically significant (t test, *p* = 0.002).

**Fig. 2:**
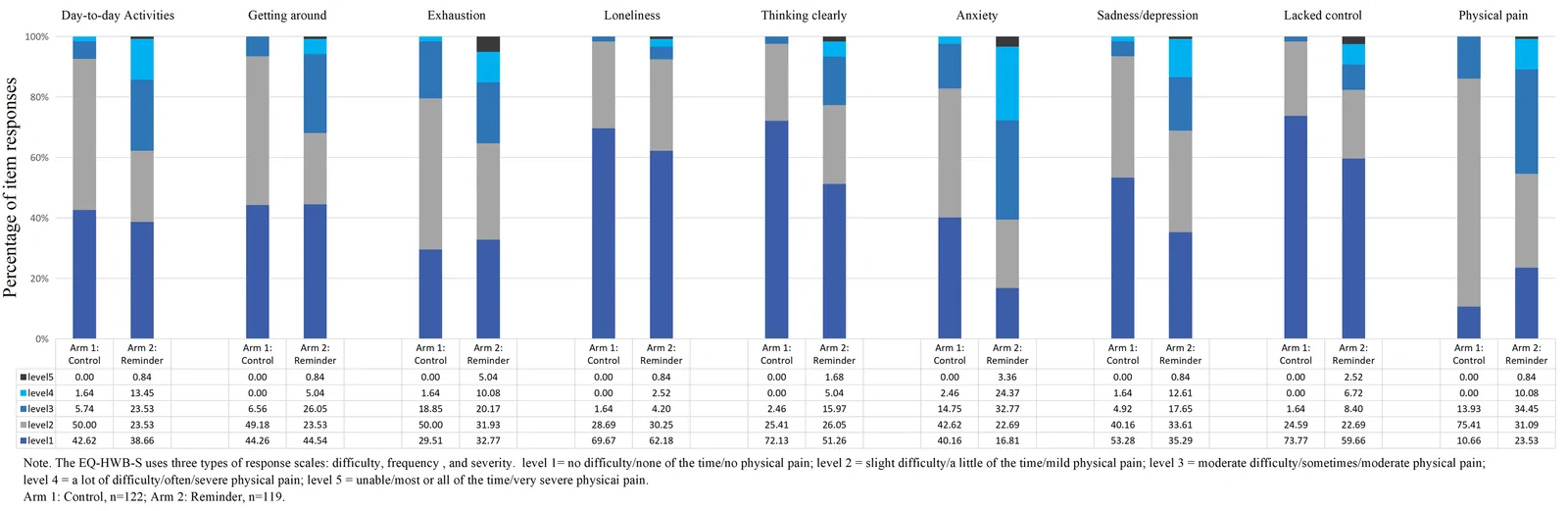
EQ-HWB-9 responses between two arms

Figure 2 presents the response distributions of the EQ-HWB-9 items for the control group (Arm 1, standard “previous seven days” recall) and the intervention group (Arm 2, symptom recall with reference to a healthy time).

Consistent with the EQ-5D-5L results, several EQ-HWB-9 items show no responses at levels 4 or 5 in the control group, indicating limited use of the upper response categories under the standard recall instruction. In contrast, responses at levels 4 and 5 are observed in the intervention group across multiple items, reflecting broader use of the response scale following the intervention.

For the physical items—day-to-day activities, getting around, exhaustion, and pain—differences between the two arms are mainly observed at intermediate and higher severity levels. For day-to-day activities, getting around, and exhaustion, the proportion of level-1 responses is similar between the two arms; however, in the control group responses are concentrated at level 2, whereas in the intervention group a substantial proportion of these level-2 responses is redistributed toward more severe levels (levels 3–5). Pain follows a similar redistribution pattern, but differs from the other physical items in two respects: first, the proportion of level-1 responses differs between the two arms; and second, level 2 represents the most frequently selected response category for pain in the control group, but not in the reminder group, where level 3 is most frequent, showing divergent clustering peaks across study arms.

In contrast, the reduction of ceiling effects is most evident for the cognitive and psychosocial items, including cognition, anxiety, sadness, and sense of control. For these dimensions, Arm 2 shows a clear decrease in level-1 responses accompanied by a broader spread of responses across higher severity levels, closely mirroring the redistribution observed for the anxiety/depression dimension of the EQ-5D-5L. Loneliness is the only exception to this overall pattern, with similar response distributions observed between the two arms and no clear evidence of redistribution toward higher severity levels. Fisher’s exact tests showed that response distributions differed significantly between the control and reminder arms for eight of the nine EQ-HWB-9 items (all *p* < 0.01). The only exception was the EQ-HWB-9 loneliness item, for which the difference was not statistically significant (*p* = 0.214).

Table 2 presents the adjusted ordered logistic regression results for the EQ-5D-5L dimensions. After adjustment for sociodemographic and clinical covariates, the pre-survey reminder was consistently associated with higher reported problem severity across all five EQ-5D-5L dimensions. Compared with respondents who received no reminder, those in the intervention group had significantly higher odds of reporting more severe problems in mobility (OR = 2.18, *p* < 0.01), self-care (OR = 3.13, *p* < 0.001), usual activities (OR = 2.78, *p* < 0.001), pain/discomfort (OR = 1.90, *p* < 0.05), and anxiety/depression (OR = 2.51, *p* < 0.001).

**Table 2:** Adjusted ordered logistic regression results for EQ-5D-5L, with reminder as the primary exposure

Variable	Mobility OR (SE)	Self-care OR (SE)	Usual activities OR (SE)	Pain/ discomfort OR (SE)	Anxiety/ depression OR (SE)
Primary exposure					
Reminder (Ref: no reminder)	2.180^**^(0.585)	3.126^***^(0.858)	2.783^***^(0.763)	1.901^*^(0.498)	2.511^***^(0.652)
Sociodemographic covariates					
Sex: female	1.213(0.330)	1.385(0.377)	1.193(0.326)	1.160(0.306)	1.125(0.289)
Age: 41–60 years	0.886(0.355)	0.969(0.393)	1.103(0.454)	0.933(0.352)	1.893(0.711)
Age: >60 years	1.545(0.653)	1.940(0.829)	1.623(0.699)	1.181(0.466)	1.518(0.605)
Education: high school / vocational school	0.798(0.314)	0.875(0.358)	0.831(0.329)	0.934(0.359)	1.081(0.411)
Education: college / university	0.900(0.350)	0.987(0.386)	0.754(0.299)	0.951(0.354)	0.526(0.193)
Clinical covariates					
Diagnosis: Structural disease	1.244(0.385)	1.437(0.449)	1.029(0.325)	0.993(0.305)	1.012(0.300)
Diagnosis: other / non-hepatobiliary	1.002(0.421)	0.962(0.405)	0.843(0.358)	1.335(0.525)	0.905(0.352)
Chief complaint: non–abdominal pain dominant	0.339^***^(0.095)	0.292^***^(0.084)	0.278^***^(0.080)	0.243^***^(0.068)	0.395^***^(0.104)
Chronic condition: yes	1.021(0.274)	1.215(0.324)	1.240(0.334)	0.918(0.240)	1.054(0.268)
Nagelkerke R^2^, full model	0.132	0.186	0.174	0.153	0.148
Δ Nagelkerke R² for reminder	0.039	0.076	0.062	0.026	0.055

***p < 0.001, **p < 0.01, *p < 0.05

Main complaint type was also strongly associated with EQ-5D-5L outcomes. Participants presenting with non–abdominal pain–dominant complaints had substantially lower odds of reporting more severe problems across all five dimensions compared with those with abdominal pain–dominant complaints (all *p* < 0.001). In contrast, sex, age group, educational attainment, diagnosis category, and history of chronic conditions were not significantly associated with problem severity for most dimensions.

Model fit was moderate across dimensions, with Nagelkerke R² values ranging from 0.13 to 0.19. The additional explanatory contribution of the reminder, assessed using Δ Nagelkerke R², ranged from 0.026 for pain/discomfort to 0.076 for self-care. Overall, these results indicate that the reminder intervention was independently associated with increased reporting of health problems across both physical and mental dimensions of the EQ-5D-5L.

Table 3 presents the adjusted ordered logistic regression results for the EQ-HWB-9 items. After adjustment for sociodemographic and clinical covariates, the pre-survey reminder was significantly associated with higher reported problem severity for most EQ-HWB-9 items. Strong and statistically significant associations were observed for day-to-day activities (OR = 2.54, *p* < 0.001), getting around (OR = 1.94, *p* < 0.05), thinking clearly (OR = 3.13, *p* < 0.001), anxiety (OR = 5.43, *p* < 0.001), sadness or depressed mood (OR = 2.78, *p* < 0.001), perceived lack of control (OR = 2.29, *p* < 0.01), and physical pain (OR = 2.40, *p* < 0.001). No statistically significant association was observed for exhaustion or loneliness.

**Table 3:** Adjusted ordered logistic regression results for EQ-HWB-9, with reminder as the primary exposure

Variable	Day-to-day activities OR (SE)	Getting around OR (SE)	Exhaustion OR (SE)	Loneliness OR (SE)	Thinking clearly OR (SE)	Anxiety OR (SE)	Sadness/ depressed OR (SE)	Lacked control OR (SE)	Physical pain OR (SE)
Primary exposure									
Reminder (Ref: no reminder)	2.542^***^(0.656)	1.940^*^(0.501)	1.282 (0.316)	1.595 (0.452)	3.129^***^(0.879)	5.429^***^(1.461)	2.778^***^(0.721)	2.289^**^(0.645)	2.398^***^(0.633)
Sociodemographic covariates									
Sex: female	1.255 (0.325)	1.260 (0.329)	1.623 (0.415)	1.516 (0.442)	1.523 (0.436)	1.206 (0.312)	1.230 (0.320)	1.341 (0.387)	1.273 (0.340)
Age: 41-60 years	1.346 (0.510)	1.103 (0.432)	1.304 (0.477)	1.262 (0.554)	1.642 (0.697)	1.472 (0.545)	1.368 (0.516)	1.199 (0.520)	1.357 (0.526)
Age: >60 years	1.580 (0.635)	1.232 (0.510)	1.442 (0.551)	1.780 (0.818)	2.134 (0.972)	1.093 (0.433)	0.880 (0.350)	1.733 (0.786)	1.208 (0.489)
Education: high school / vocational school	0.973 (0.367)	0.866 (0.333)	0.619 (0.222)	0.757 (0.334)	0.983 (0.419)	0.735 (0.274)	0.737 (0.289)	0.947 (0.406)	1.817 (0.696)
Education: college / university	0.740 (0.269)	0.605 (0.228)	0.805 (0.293)	0.973 (0.405)	1.432 (0.565)	0.787 (0.284)	0.895 (0.323)	1.007 (0.417)	1.419 (0.538)
Clinical covariates									
Diagnosis: Structural disease	1.479 (0.439)	1.362 (0.405)	1.066 (0.315)	0.967 (0.322)	1.057 (0.343)	1.253 (0.379)	1.027 (0.306)	1.017 (0.335)	1.390 (0.431)
Diagnosis: Other / non-hepatobiliary	1.317 (0.510)	1.467 (0.576)	0.889 (0.327)	0.824 (0.368)	0.830 (0.351)	1.467 (0.580)	1.074 (0.416)	1.014 (0.439)	1.296 （0.531）
Chief complaint: non–abdominal pain dominant	0.363^***^(0.097)	0.370^***^(0.100)	0.578^*^(0.147)	0.543^*^(0.161)	0.821 (0.229)	0.547^*^(0.145)	0.405^***^(0.109)	0.841 (0.242)	0.230^***^（0.066）
Chronic condition: yes	1.533 (0.391)	1.561 (0.402)	1.099 (0.273)	1.411 (0.401)	1.161 (0.320)	1.545 (0.402)	1.097 (0.284)	1.084 (0.306)	1.089 （0.283）
Nagelkerke R^2^, full model	0.143	0.125	0.058	0.066	0.097	0.218	0.132	0.060	0.174
Δ Nagelkerke R² for reminder	0.057	0.029	0.005	0.014	0.082	0.179	0.070	0.044	0.047

***P < 0.001, **P < 0.01, *P < 0.05

Main complaint type was again strongly associated with multiple EQ-HWB-9 outcomes. Compared with participants presenting with abdominal pain–dominant complaints, those with non–abdominal pain–dominant complaints had significantly lower odds of reporting more severe problems for day-to-day activities, getting around, exhaustion, loneliness, anxiety, sadness or depressed mood, and physical pain (all *p* < 0.05). Associations with thinking clearly and perceived lack of control were not statistically significant.

Other covariates, including sex, age group, educational attainment, diagnosis category, and history of chronic conditions, showed no consistent or systematic associations across EQ-HWB-9 items. Model fit varied by item, with Nagelkerke R² values ranging from 0.06 to 0.22, and the highest explanatory power observed for anxiety and physical pain. Δ Nagelkerke R² for the reminder ranged from 0.005 for exhaustion to 0.179 for anxiety, suggesting that the reminder contributed more to the explanation of some cognitive and emotional items than to others.

## Discussion

In this study, we examined whether a brief pre-survey reminder prompting respondents to reflect on their pre-illness health alters patient-reported outcome responses in a cross-sectional sample of hepatobiliary surgery patients. Across both the EQ-5D-5L and the EQ-HWB-9, the reminder was consistently associated with higher reported problem severity and lower EQ-VAS scores, even after adjustment for sociodemographic and clinical characteristics. Importantly, baseline characteristics were comparable between the two study arms, supporting the interpretation that the observed differences are attributable to the instructional intervention rather than differences in case mix.

At the descriptive level, the reminder was associated with a clear reduction in ceiling effects. Under the standard “today” recall instruction, responses were heavily concentrated at the lowest severity level, with little or no use of the most severe categories, particularly for the EQ-5D-5L. Following the reminder, responses were redistributed away from level 1 toward higher severity levels across all EQ-5D-5L dimensions and most EQ-HWB-9 items. This pattern was especially pronounced for pain/discomfort and anxiety/depression, as well as for cognitive and psychosocial items such as thinking clearly, sadness, and perceived lack of control. These findings suggest that the reminder encouraged broader use of the response scale and increased differentiation between severity levels.

The regression analyses further support this interpretation. After covariate adjustment, the reminder was independently associated with higher odds of reporting more severe problems across all EQ-5D-5L dimensions and across most EQ-HWB-9 items. The magnitude of these associations was substantial, particularly for self-care, usual activities, anxiety, and cognitive or emotional wellbeing domains. Notably, the direction of the association was consistent across instruments, despite differences in content and recall period, suggesting that the effect reflects a general shift in response appraisal rather than instrument-specific artefacts.

From a conceptual perspective, these findings are compatible with a recalibration-related interpretation [24]. Patients hospitalised for surgery may implicitly adjust their internal standards when rating their current health, particularly if they have adapted to symptoms or functional limitations. By prompting respondents to consider how their health was when they were not ill, the reminder appears to have re-anchored these internal standards, leading respondents to report greater problems under the same “today” recall period. Importantly, this does not imply that the reminder worsened health or induced negative reporting bias; rather, it suggests that the standard recall instruction may underestimate problem severity in cross-sectional patient samples due to unobserved recalibration. Because the EQ-5D-5L and EQ-HWB-9 differ in both recall period and item content, the present study cannot isolate whether the reminder effect varied by recall period. Nevertheless, the reminder was associated with similar directional changes across both instruments, suggesting that its influence on response appraisal may not be limited to a single recall period. Future studies using factorial designs are needed to examine whether recall period modifies the effect of such reminders.

The domain-specific patterns observed across the EQ-HWB-9 provide additional insight into how this intervention operates. While most items showed increased reported severity following the reminder, exhaustion and loneliness did not show statistically significant associations in the adjusted models. These items may be less directly linked to acute illness or surgical context, or they may rely on more stable personal reference points that are less sensitive to short cognitive prompts. In contrast, items related to pain, emotional distress, cognition, and perceived control—domains likely to be strongly affected by illness appraisal—showed the largest effects. This heterogeneity suggests that recalibration may not operate uniformly across all aspects of health and wellbeing.

The consistent association between main complaint type and outcomes across both instruments also warrants attention. Participants presenting with non–abdominal pain–dominant complaints reported lower problem severity across many dimensions, independent of the reminder. This finding likely reflects genuine differences in symptom burden and illness experience and supports the validity of the outcome measures. Importantly, diagnosis category and history of chronic conditions showed limited associations with reported severity after adjustment, suggesting that the reminder effect was not simply capturing underlying disease severity.

Methodologically, this study contributes to the response shift literature by examining whether a simple pre-survey reminder is associated with altered PROM response patterns in a cross-sectional setting. Most existing approaches to response shift rely on longitudinal designs, retrospective assessments, or complex modelling strategies that are difficult to implement in routine clinical or survey settings [11, 16–19]. In contrast, the intervention tested here is simple, low-burden, and preserves the original instrument wording and recall period. By focusing on the appraisal process at the point of data collection, this study suggests a pragmatic approach for examining how pre-survey reminders may influence PROM responses, particularly when comparing patient groups with different illness experiences.

Several limitations should be acknowledged. First, the sample was recruited from a single hepatobiliary surgery department and was not intended to be representative of all hepatobiliary surgery patients or broader patient populations. The findings should therefore be interpreted as exploratory. Future studies should examine whether similar reminder-related response patterns are observed in other hospitals, clinical specialties, outpatient settings, and more diverse patient samples. Second, the design involved two independent samples rather than randomisation at the individual level, although baseline comparability between groups reduces concerns about confounding. Third, while the findings are compatible with a recalibration-related interpretation, the study does not directly measure cognitive processes underlying appraisal, and alternative explanations—such as increased symptom salience induced by the reminder—cannot be fully excluded. In addition, the study did not include an objective benchmark for true health status, so it cannot determine whether the reminder corrected bias or instead increased symptom salience, induced a framing effect, or triggered another response process. Finally, the sample was recruited from a single hepatobiliary surgery department and was not intended to be demographically representative of the broader patient population in China. The findings should therefore be interpreted as exploratory and may not generalise to other patient groups. In addition, the reminder asks patients to reflect on their health when they did not have the current health condition, which may introduce recall-related bias. Its effect may vary according to the type, duration, and impact of the health condition, as well as patient characteristics such as age. The approach may also be less suitable for patients with cognition-related conditions or memory problems, for whom recalling pre-illness health may be difficult or unreliable. Future studies should examine the reminder in broader populations and assess whether its effect differs across clinical conditions and patient subgroups.

Despite these limitations, the results have important implications for the use of generic PROMs in cross-sectional clinical research. When instruments such as the EQ-5D are used in cross-sectional studies, respondents’ frames of reference may influence how they report current health. Simple pre-survey reminders, such as the one tested here, may affect these appraisal processes and response patterns. However, the mechanism underlying the reminder-related response pattern remains uncertain. A key unresolved question is whether responses under the standard instruction or responses after the pre-illness reference reminder more closely reflect patients’ underlying actual health status. Future studies using think-aloud interviews, cognitive debriefing, and objective or clinician-rated indicators of health status would help clarify how patients interpret the reminder and whether it improves the accuracy of PROM responses.

## Conclusion

In conclusion, this study provides empirical evidence that a brief pre-survey reminder referencing pre-illness health can meaningfully influence patient-reported outcome responses in a cross-sectional surgical sample. The findings highlight the importance of appraisal processes in PROM data and suggest that pre-illness reference reminders may alter response patterns in routine data collection. Further research is needed to assess the generalisability of this approach across settings, populations, and instruments, and to evaluate its implications for longitudinal assessment and health economic evaluation.

## Data Availability

The datasets generated and analyzed during the current study are not publicly available in order to protect participant privacy. De-identified data are available from the corresponding author on reasonable request.
